# Predicting DNA-binding sites of proteins from amino acid sequence

**DOI:** 10.1186/1471-2105-7-262

**Published:** 2006-05-19

**Authors:** Changhui Yan, Michael Terribilini, Feihong Wu, Robert L Jernigan, Drena Dobbs, Vasant Honavar

**Affiliations:** 1 Department of Computer Science, Utah State University, Logan, Utah, 84341, USA; 2 Department of Genetics, Development and Cell Biology, Iowa State University, Ames, Iowa, 50010, USA; 3 Bioinformatics and Computational Biology Graduate Program, Iowa State University, Ames, Iowa, 50010, USA; 4 Artificial Intelligence Research Laboratory, Iowa State University, Ames, Iowa, 50010, USA; 5 Department of Computer Science, Iowa State University, Ames, Iowa, 50010, USA; 6 Center for Computational Intelligence, Learning, and Discovery, Iowa State University, Ames, Iowa, 50010, USA; 7 Laurence H Baker Center for Bioinformatics and Biological Statistics, Iowa State University, Ames, Iowa, 50010, USA; 8 Department of Biochemistry, Biophysics, and Molecular Biology, Iowa State University, Ames, Iowa, 50010, USA

## Abstract

**Background:**

Understanding the molecular details of protein-DNA interactions is critical for deciphering the mechanisms of gene regulation. We present a machine learning approach for the identification of amino acid residues involved in protein-DNA interactions.

**Results:**

We start with a Naïve Bayes classifier trained to predict whether a given amino acid residue is a DNA-binding residue based on its identity and the identities of its sequence neighbors. The input to the classifier consists of the identities of the target residue and 4 sequence neighbors on each side of the target residue. The classifier is trained and evaluated (using leave-one-out cross-validation) on a non-redundant set of 171 proteins. Our results indicate the feasibility of identifying interface residues based on local sequence information. The classifier achieves 71% overall accuracy with a correlation coefficient of 0.24, 35% specificity and 53% sensitivity in identifying interface residues as evaluated by leave-one-out cross-validation. We show that the performance of the classifier is improved by using sequence entropy of the target residue (the entropy of the corresponding column in multiple alignment obtained by aligning the target sequence with its sequence homologs) as additional input. The classifier achieves 78% overall accuracy with a correlation coefficient of 0.28, 44% specificity and 41% sensitivity in identifying interface residues. Examination of the predictions in the context of 3-dimensional structures of proteins demonstrates the effectiveness of this method in identifying DNA-binding sites from sequence information. In 33% (56 out of 171) of the proteins, the classifier identifies the interaction sites by correctly recognizing at least half of the interface residues. In 87% (149 out of 171) of the proteins, the classifier correctly identifies at least 20% of the interface residues. This suggests the possibility of using such classifiers to identify potential DNA-binding motifs and to gain potentially useful insights into sequence correlates of protein-DNA interactions.

**Conclusion:**

Naïve Bayes classifiers trained to identify DNA-binding residues using sequence information offer a computationally efficient approach to identifying putative DNA-binding sites in DNA-binding proteins and recognizing potential DNA-binding motifs.

## Background

Protein-DNA interactions play a pivotal role in gene regulation. The ability to identify amino acid residues that are responsible for the specificity and affinity of the interactions can significantly improve our understanding of macromolecular functions and contribute to advances in drug discovery [1,2]. Hence, the discovery of the principles of protein-DNA interactions has been a topic of significant interest for many years [3]. Current approaches to uncovering such principles rely on experimental analysis of the structures of protein-DNA complexes in order to understand the molecular details of specific residue-residue contacts that mediate protein-DNA recognition [4-6]. In addition to biophysical methods for structure determination, biochemical and molecular genetic approaches have been widely used to identify DNA-binding sites on proteins and to investigate the interaction modes between proteins and DNA. For example, alanine-scanning mutagenesis has been used to identify the amino acids important for target recognition by the m^5^C methyltransferase [7] and to distinguish specific amino acids important for DNA binding and transcription activation by SoxS [8]. More recently, methods for precisely identifying protein-DNA contacts by coupling photochemical crosslinking with mass spectrometry have also been developed [9].

With increasing availability of protein sequence data, there is an urgent need for computational tools that can rapidly and reliably identify DNA-binding sites. Hence, there has been significant recent interest in developing computational methods for identification of amino acid residues that participate in protein-DNA interactions based on combinations of sequence, structure, evolutionary information, and chemical or physical properties. For example, Jones *et al. *[10] analyzed residue patches on the surface of DNA-binding proteins and used electrostatic potentials of residues to predict DNA-binding sites. They recently applied this method to the identification of three specific classes of DNA-binding proteins, based on the presence of solvent accessible DNA-binding structural motifs [11]. In related work, Tsuchiya *et al. *[12] used a structure-based method to identify protein-DNA binding sites based on electrostatic potentials and surface shape, and Keil *et al*. [13] trained a neural network classifier to identify patches likely to be DNA-binding sites based on physical and chemical properties of the patches. Neural network classifiers have also been used to identify protein-DNA interface residues based on a combination of sequence neighbor and structure information [14]. More recently, Ahmad and Sarai have proposed a sequence-based method for predicting DNA-binding residues that incorporates sequence alignment profiles into the input [15].

Against this background, this paper describes a machine-learning approach to developing a classifier for identifying amino acid residues that are likely to be involved in protein-DNA interactions.

## Results

### Identification of interface residues based on local sequence information

A Naïve Bayes classifier was trained to predict whether or not a target residue in a protein sequence is an interface residue based on local protein sequence information. Several input encodings based on local sequence information were tried, with input consisting of: (a) the identities of 9 amino acid residues, corresponding to a window containing the target residue and 4 neighboring residues on each side of the target residue; and (b) the identities of 9 amino acid residues and the sequence entropy of the target residue (the entropy of the corresponding column in multiple alignment obtained by aligning the target sequence with its sequence homologs). In each case, Naïve Bayes classifiers were trained and evaluated using leave-one-out cross-validation on a set of 171 DNA-binding proteins

Table 1 shows that the classifier using amino acid identities as input achieved an overall accuracy of 71% with a correlation coefficient of 0.24, 35% of the residues predicted to be interface residues are actually interface residues, and 53% of interface residues are correctly identified. Adding the sequence entropy of the target residue (the entropy of the corresponding column in multiple alignment obtained by aligning the target sequence with its sequence homologs) to the input improved the performance of the classifier (Table 1). The resulting classifier achieved an overall accuracy of 78% with a correlation coefficient of 0.28, 44% specificity, and 41% sensitivity. In 33% (56 of 171) of the proteins, the classifier recognizes the interaction site by correctly identifying at least half of the interface residues, and in 87% (149 of 171) of the proteins, by correctly identifying at least 20% of the interface residues.

Inclusion of other features of the target residue, including relative solvent accessibility, secondary structure, electrostatic potential, and hydrophobicity as additional inputs to the classifier did not yield performance improvements (data not shown) relative to the classifier trained using only the amino acid identities of the target residue and its sequence neighbors. Classifiers trained using features other than the amino acid identities of target residue and its neighbors as input achieved performance that was lower than that of the classifier using amino acid identities of the corresponding residues as input (data not shown).

### Evaluation of the predictions in the context of 3-dimensional structures of proteins

**Figure 1:**
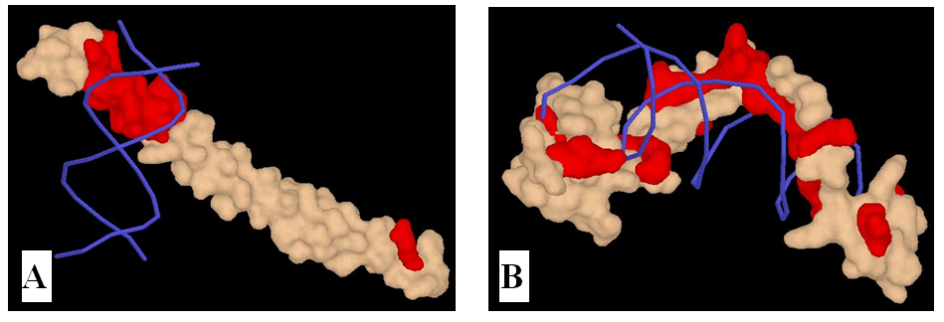
**Visualization of predicted DNA-binding residues on 3-D Structure**. The predicted interface residues are shown in red on protein surface. DNA molecules bound to the proteins are shown in blue. **A**: The predictions on C/Ebpβ from PDB complex 1gu4, the 3^rd ^best out of the 179 proteins in terms of correlation coefficient. **B**: The predictions on I-*Tev*I from PDB complex 1i3j, the 114^th ^best out of the 179 proteins. Figures are generated using Protein Explorer [38].

We examined in the context of the 3-dimensional structures of the protein-DNA complexes, the DNA-binding residue predictions generated by a Naïve Bayes classifier trained to identify such residues based on the amino acid identities of the target residue and its sequence neighbors. Two representative examples are shown in figure 1. Figure 1A shows the predictions on the transcription factor C/Ebpβ from PDB complex 1gu4. The predictions of the classifier rank the 3^rd ^best in terms of correlation efficient among the 171 proteins. We note that the classifier is able to recognize the DNA-binding site on the protein on the basis of sequence information alone. Figure 1B shows the predictions on the intron-associated endonuclease I-*Tev*I from PDB complex 1i3j. The predictions of the classifier in this case rank the114^th ^best among the 171 proteins in terms of correlation efficient. I-*Tev*I wraps around the DNA and has an unusually extended binding site. We note that the predicted DNA-binding residues cover the long segment of the protein that binds to the DNA.

### Receiver operating characteristic (ROC) curve

**Figure 2:**
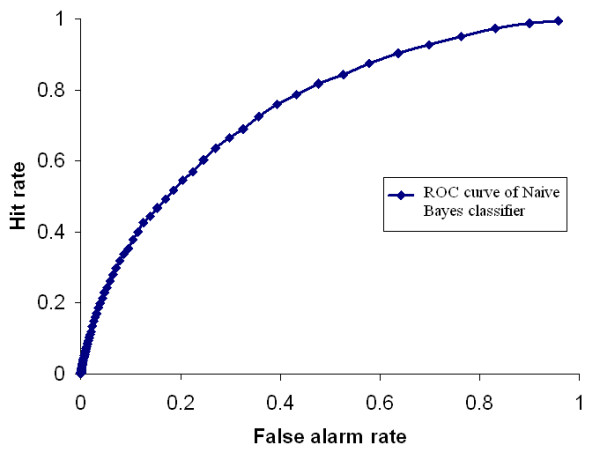
Receiver Operating Characteristic curve (ROC curve) for interface residue identification.

In some situations (e.g., identification of critical interface residues for site-specific mutagenesis), it is desirable to predict interface residues with high precision at the cost of reduced coverage. In other situations, discovering more potential interface residues might be more useful. These different requirements can be met by modifying the threshold θ used by the Naïve Bayes classifier in this study. The Naïve Bayes classifier predicts a residue to be an interface residue if P(c=1|X=x1x2...xn)P(c=0|X=x1x2...xn)>θ
 MathType@MTEF@5@5@+=feaafiart1ev1aaatCvAUfKttLearuWrP9MDH5MBPbIqV92AaeXatLxBI9gBaebbnrfifHhDYfgasaacH8akY=wiFfYdH8Gipec8Eeeu0xXdbba9frFj0=OqFfea0dXdd9vqai=hGuQ8kuc9pgc9s8qqaq=dirpe0xb9q8qiLsFr0=vr0=vr0dc8meaabaqaciaacaGaaeqabaqabeGadaaakeaadaWcaaqaaiabdcfaqjabcIcaOiabdogaJjabg2da9iabigdaXiabcYha8jabdIfayjabg2da9iabdIha4naaBaaaleaacqaIXaqmaeqaaOGaemiEaG3aaSbaaSqaaiabikdaYaqabaGccqGGUaGlcqGGUaGlcqGGUaGlcqWG4baEdaWgaaWcbaGaemOBa4gabeaakiabcMcaPaqaaiabdcfaqjabcIcaOiabdogaJjabg2da9iabicdaWiabcYha8jabdIfayjabg2da9iabdIha4naaBaaaleaacqaIXaqmaeqaaOGaemiEaG3aaSbaaSqaaiabikdaYaqabaGccqGGUaGlcqGGUaGlcqGGUaGlcqWG4baEdaWgaaWcbaGaemOBa4gabeaakiabcMcaPaaacqGH+aGpcqaH4oqCaaa@5936@. Figure 2 shows the Receiver Operating Characteristic curve (ROC curve) of the DNA-binding site predictor.

### Naïve Bayes classifier using only local sequence identities as input can discover DNA-binding motifs

**Figure 3:**
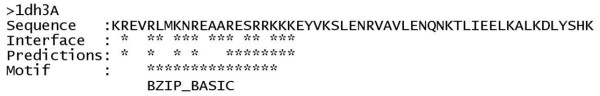
**Comparison of actual and predicted DNA-binding site residues for transcription factor CREB (PDB 1dh3A)**. PROSITE motif BZIP_BASIC (bottom row) covers many of the actual interface residues (the first row below sequence). Note that the predictions of Naïve Bayes classifier (the second row below sequence) overlap with the PROSITE motifs, but more closely correspond to the actual interface residues.

The results summarized above show that a Naïve Bayes classifier trained on a set of DNA-binding proteins can successfully identify protein-DNA interface residues from amino acid sequence. This raises the question as to how the sequence features that are identified as predictive of DNA-binding residues by Naïve Bayes classifier relate to known DNA-binding motifs. To explore this question, we used the ps_scan program to search for PROSITE motifs in our data set of 171 DNA-binding proteins. PROSITE motifs were found in 53 of the 171 proteins (a total of 73 hits). Of these 73 hits, 61 overlap with actual protein-DNA binding sites. The DNA-binding site predictions produced by the Naïve Bayes classifier (in the leave-one-out cross-validation setting) using the identities of a window of 9 residues and the sequence entropy of the target residue as input, substantially overlap with 56 of the 61 PROSITE DNA-binding motifs (Figure 3). It is worth noting that 118 of the 171 DNA-binding proteins in our data set contain *no *PROSITE motif whose annotation suggests a role in protein-DNA interactions. PROSITE motifs cover more than 50% of interface residues in only 11% (18 out of 171) of the proteins and cover at least 20% of interface residues in only 20% (34 out of 171) of the proteins. In contrast, the Naïve Bayes classifier identifies at least 50% of the interface residues in 33% (56 out of 171) of the proteins and at least 20% of the interface residues in 87% (149 out of 171) of the DNA-binding proteins used in this study. These results suggest the possibility of using a Naïve Bayes classifier trained to predict DNA-binding residues to identify putative DNA-binding motifs.

### Comparison with previously published methods

**Figure 4:**
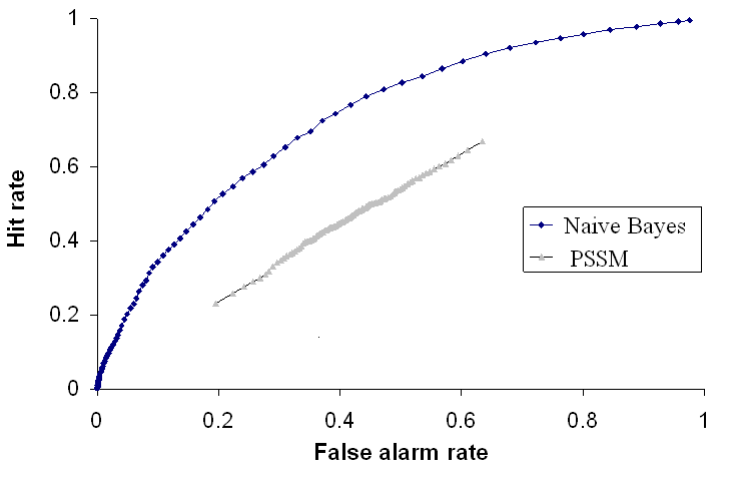
**The ROC curves for the Naïve Bayes classifier and the PSSM-based classifier**. The Naïve Bayes classifier uses the identities of 9 amino acid residues as input. The ROC for the Naïve Bayes classifier is obtained using Weka on 86 DNA-binding proteins with lengths ranging from 40 to 200 residues with pairwise sequence similarity less than 30%. The ROC for the PSSM-based classifier is generated using the true positive, false positive, true negative, and false negative predictions obtained by submitting the 86 sequences to the online server [16] that implements PSSM-based classifier developed by Ahmad and Sarai [15].

Ahmad and Sarai have developed a Position Specific Scoring Matrix (PSSM) based neural network classifier for predicting DNA-binding sites [15]. To the best of our knowledge, this is the only previously published study which reports the performance of a DNA-binding site prediction using only sequence information on a "per residue" basis. Ahmad and Sarai have made available an online server that predicts DNA-binding residues using a PSSM-based neural network classifier [16]. The server makes predictions for protein sequences that are 40 to 200 amino acid residues in length. In our data set of 171 DNA-binding proteins, 86 have length in this range. The predictions of the PSSM-based classifier on these 86 proteins were obtained by submitting the sequences to the online server. The server returns, for each residue in the submitted sequence, the estimated probability that the residue is a DNA-binding residue. These probabilities can be compared with a threshold to obtain a prediction as to whether a residue is a DNA-binding residue. Different choices of threshold yield different predictions. We varied the threshold from 0.01 to 0.99 in increments of 0.02 to generate an ROC curve for the PSSM-based neural network classifier. For comparison, we trained and evaluated using leave-one-out cross-validation, a Naïve Bayes classifier using as input the identities of 9 amino acid residues on the subset of 86 proteins (ranging from 40 to 200 amino acids in length). Figure 4 shows the comparison of the ROC curves of the PSSM-based neural network classifier with that of the Naïve Bayes classifier on the data set of 86 proteins. The results show that the Naïve Bayes classifier achieves higher hit rate, for any given choice of the false alarm rate, than the current implementation of the PSSM-based neural network classifier in the online server.

### Identification of DNA-binding residues in type I restriction-modification system

**Figure 5:**
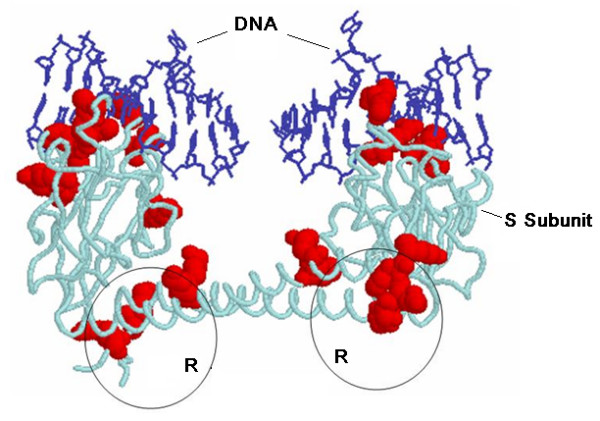
**The predictions on the S subunit of the type I (R-M) system from *M. jannaschi***. The predicted interface residues are shown in red. The DNA molecules from the interaction model proposed by Kim *et al. *[17] are shown in blue. The locations of R units in Kim's model are indicated by circles. Figures are generated using Protein Explorer [38].

Restriction-modification (R-M) systems play important role in the recognition and elimination of foreign DNA. In type I R-M systems, S subunit determines the specificity of DNA recognition. The interaction mode between S subunit and DNA is still unknown. Recently, Kim *et al. *[17] solved the crystal structure of the S subunit from *M. jannaschii*, the only crystal structure ever reported for the S subunit of type I (R-M) systems. To further evaluate the Naïve Bayes classifier, we used the classifier trained on our data set of 171 DNA-binding proteins (using identities of the target residue, and 4 sequence neighbors on either side along with the sequence entropy of the target residue as input) to identify DNA-binding residues on the S subunit of the type I R-M system from *M. jannaschii*. Figure 5 shows the predicted DNA-binding residues in red and spacefill. Note that Kim *et al. *[17] reported, based on the solved crystal structure of the S subunit of *M. jannaschii*, that the structures of the two target recognition domains (TRD1, residue 1–168 and TRD2, residue 209–378) of the S subunit are similar to the DNA binding domain of *Taq*I-MTase. By aligning the structures of TRD1 and TRD2 with the structure of *Taq*I-MTase/DNA complex, Kim *et al. *[17] proposed a model for the interaction between the S subunit and DNA. In figure 5, the DNA molecules in Kim's model are shown in blue. Comparison of Kim's model with the DNA-binding site predictions produced by our Naïve Bayes classifier shows that the Naive Bayes classifier agrees with the locations of the two potential DNA-binding sites on the S subunit in Kim's interaction model.

Figure 5 also shows that two additional DNA-binding sites predicted by the Naïve Bayes classifier overlap with the potential interaction sites between the S subunit and R subunits of the protein (shown as circles in figure 5) as proposed in Kim's model. This observation raises the intriguing possibility that protein-DNA interfaces and protein-protein interfaces might have some common features.

### Predictions of the Naïve Bayes classifier on proteins for which there is no experimental evidence suggesting a DNA-binding role

Given that the Naïve Bayes classifier was trained to identify DNA-binding residues in proteins that are known to bind to DNA, it is interesting to examine their predictions on a set of proteins for which at present, there is no evidence suggesting a DNA-binding role. We assembled a non-redundant data set of 2,323 proteins which, based on our analysis of Gene Ontology annotations, appear to have no evidence suggesting a DNA-binding role. A Naïve Bayes classifier trained on our data set of 171 DNA-binding proteins to identify the DNA-binding residues (using amino acid identities of the target residue and its sequence neighbors together with the sequence entropy of the target residue as input) was applied to the 2,323 proteins with no known DNA-binding role. The Naïve Bayes classifier predicted 11% of the 613,754 residues from these 2,323 proteins as potentially DNA-binding residues. It would be inappropriate to conclude that 11% is a per residue basis false positive rate of our classifier because absence of DNA-binding evidence in GO annotation does not necessarily imply that the protein in question does not have a DNA-binding role. It is quite possible that at least some of these 2,323 proteins indeed bind to DNA. It should be emphasized that our classifier was *not *trained to distinguish the class of DNA-binding proteins from those that are not DNA-binding (Training such a classifier would involve using representatives of both DNA-binding and non DNA-binding proteins in the training set). It is interesting to note that in 156 of the 2,323 proteins, *no *residues were predicted to be DNA-binding by our classifier; 264 had fewer than 5 predicted DNA-binding residues; 502 had fewer than 10 predicted DNA-binding residues, and 999 with fewer than 20 DNA-binding residues. Exploring the implications of these observations would require experimentally testing some of the proteins on which our Naïve Bayes classifier predicts putative DNA-binding sites for DNA-binding activity. Another potentially interesting direction would be to train classifiers to distinguish proteins that are DNA-binding (without necessarily identifying the DNA-binding residues) from those that are not.

## Discussion

### Effectiveness of local amino acid sequence based approach to prediction of putative DNA-binding sites

In this paper, we have described a computationally efficient approach to identifying putative DNA-binding residues of DNA-binding proteins using Naïve Bayes classifiers trained to predict DNA-binding residues using amino acid identities of the target residue and its sequence neighbors. The resulting classifier achieves 71% overall accuracy with a correlation coefficient of 0.24, 35% specificity and 53% sensitivity in identifying interface residues as evaluated by leave-one-out cross-validation. Our results indicate the feasibility of identifying interface residues based on local sequence information alone.

We found that the performance of the classifier is improved by using sequence entropy of the target residue (the entropy of the corresponding column in multiple alignment obtained by aligning the target sequence with its sequence homologs) as additional input. This observation is consistent with the suggestion that DNA-binding residues are likely to be conserved (because of their function). The resulting classifier achieves 78% overall accuracy with a correlation coefficient of 0.28, 44% specificity and 41% sensitivity in identifying interface residues.

Incorporating additional structure-derived information such as solvent accessibility, electrostatic potential, hydrophobicity or secondary structure of the target residue as additional input, however, did not improve the performance in this study. This should not be taken to mean that these features are not useful predictors of a residue's functionality. In particular, electrostatic potential has been shown to be useful in identification of protein-DNA interface residues [10,11]. The fact that this information does not improve performance of our Naïve Bayes classifiers might have to do with the properties of input encoding or the classification method. Specifically, the additional features were simply added as additional input. The underlying assumption of the Naïve Bayes classifier that the inputs are independent given the class almost certainly does not hold in the case of protein sequences. Hence, more systematic analysis is needed to identify features that are useful for identification of interface residues and develop methods of representing them in input to a broad range of classifiers. Jones and Thornton [18] analyzed six features of surface patches in protein-protein interaction sites and developed an approach to identify protein-protein interfaces based on the scores combining the six features. Sen *et al. *[19] developed an ensemble method to identify protease-inhibitor binding sites based on sequence, structure and evolution information. It would be interesting to explore such methods for computational prediction of protein-DNA interfaces.

### Comparison of Naïve Bayes classifier with a PSSM-based neural network classifier

Ahmad and Sarai [15] used a PSSM-based neural network classifier to identify interface residues in protein-DNA interactions. Our comparison of the PSSM-based classifier with the Naïve Bayes classifier shows that the Naïve Bayes classifier achieves higher hit rate than the PSSM-based classifier for any given choice of the false alarm rate.

We note that the PSSM-based classifier's ROC originally reported by Ahmad and Sarai [15] is better than the PSSM-based classifier's ROC achieved by their online server [16] on the data set used in our comparison. A few factors may have contributed to this difference: (1) the data set used by Ahmad and Sarai in their original study is different from the data set of 86 proteins used here. It is possible that the current implementation of the PSSM-based method is well optimized for their original data set, but not for the 86 proteins used here; (2) the ROC reported by Ahmad and Sarai includes predictions on proteins of all lengths, whereas the online server only makes predictions for proteins with a length in the range of 40–200. We chose to compare the Naïve Bayes classifier with the online server because the server is publicly available and it provides the raw probabilities of the predictions making it possible to compare the ROC curves of the two classifiers on the same data set. However, it should be noted that in the case of Naïve Bayes classifier, our use of leave-one-out cross-validation ensures that the training and test data do not overlap. We have no control over the training data used by the PSSM-based classifier. Nevertheless, a comparison of the two ROC curves suggests that the Naïve Bayes classifier achieves higher hit rate than the current implementation of the PSSM-based neural network classifier for any given choice of the false alarm rate.

A thorough assessment of the performance of the Naïve Bayes classifier relative to the PSSM-based classifier requires systematic comparisons using leave-one-out cross-validation on identical data sets – which is at present, not feasible without access to an implementation of the algorithm and the precise parameter settings used to train the PSSM-based classifier. Plans are underway to perform such a comparison using identical data sets and evaluation procedures, in collaboration with Ahmad and Sarai.

It should be noted that the Naïve Bayes classifier described in this paper offers several advantages over the PSSM-based neural network classifier: (a) The Naïve Bayes classifier can be trained in a single pass through the training data whereas training a neural network classifier requires many, often hundreds of passes through the training data. (b) Training the Naïve Bayes classifier, unlike the neural network classifier, requires no time-consuming and computationally expensive exploration of many possible choices of network architecture (e.g., number of hidden neurons) and parameter settings (e.g., learning rate). (c) The Naïve Bayes classifier, as well as predictions generated by it is amenable to a straightforward probabilistic interpretation whereas the neural network classifier is more of a "black box".

These advantages, together with the superior performance of the Naïve Bayes classifier relative to the current implementation of the PSSM-based neural network classifier, make it an attractive alternative to the latter in identifying DNA-binding residues from a protein sequence. However, the neural network classifier is not limited by the strong independence assumption of the Naïve Bayes classifier. Hence, it would be interesting to explore whether a neural network classifier or a variant of it could be optimized to yield results that are better than that of the simple Naïve Bayes classifier.

### Use of Naïve Bayes classifiers to identify putative novel DNA-binding motifs

Protein sequence motifs (defined here as sequence segments associated with specific protein functions or structural families) are often used to identify putative DNA-binding domains. Discovery of such motifs requires alignment of protein sequences that are known to have the same or similar functions. Generating multiple sequence alignments that reveal useful sequence motifs requires significant human expertise to identify a suitable set of sequences to be aligned and to manually refine, through an iterative process of trial and error, the multiple sequence alignment. Against this background, it is interesting to note that in 118 out of 171 DNA-binding proteins used in this study, we found *no *PROSITE motifs whose annotations suggest a possible DNA-binding role. In the remaining proteins, 61 PROSITE motifs were found to overlap with protein-DNA binding sites. The DNA-binding sites predicted by the Naïve Bayes classifier significantly overlapped with 56 of the 61 PROSITE motifs that overlapped with DNA-binding sites. PROSITE motifs cover at least 20% of the DNA-binding residues in only 20% (34 out of 171) of the proteins. In contrast, the Naïve Bayes classifier identifies at least 20% of the interface residues in 87% (149 out of 171) of the DNA-binding proteins used in this study. This raises the possibility of identifying novel sequence motifs that correspond to protein-DNA interfaces by using a Naïve Bayes classifier trained to identify protein-DNA binding sites. More systematic comparison of this approach with alternative approaches to identification of putative DNA-binding motifs using other motif libraries and different motif finding methods is needed to evaluate its efficacy relative to other approaches.

## Conclusion

In previous work, we have used similar approaches to identify interface residues involved in protein-protein interactions [20,21] and protein-RNA interactions [22]. Here we show that it is also feasible to identify interface residues involved in protein-DNA interaction using sequence information. With the level of success achieved in this study, putative DNA-binding sites predicted by the classifiers trained using a machine-learning approach should be useful for guiding experimental investigations into the role of specific residues of a protein in its interaction with DNA, e.g., by localizing candidate residues for alanine-scanning mutagenesis [7,8]. Moreover, analysis of the binding site "rules" generated by classifiers may provide valuable insight into the protein-DNA recognition code responsible for the specificity and affinity of protein-DNA interactions in living cells.

## Methods

### Data sets

DNA-binding proteins: A data set of DNA-binding proteins was extracted from structures of known protein-DNA complexes in the Protein Data Bank [23]. The dataset was culled using PISCES [24]. The resulting dataset consists of 171 proteins with mutual sequence identity <= 30% and each protein has at least 40 amino acid residues. All the structures have resolution better than 3.0 Å and R factor less than 0.3.

Proteins that do not have evidence of a DNA-binding role: A non-redundant set of proteins with mutual identity less than 30% was extracted from the PDB using the cluster file from the Protein Data Bank [25]. Structures with resolution worse than 2.5 Å were removed. The annotations for each protein were retrieved from the Gene Ontology Annotation (GOA) [26]. Proteins with annotations indicative of a DNA-binding role were eliminated, leaving a data set of 2,313 proteins with no evidence of a DNA-binding role.

### Definition of interface residues

Interface residues are defined as described in Jones *et al. *[10]. Accessible surface area (ASA) was computed for each residue in the unbound protein (in absence of DNA) and in the protein-DNA complex using NACCESS [27]. A residue is defined to be an interface residue if its ASA in the protein-DNA complex is less than its ASA in the unbound protein by at least 1Å^2^. The 171 proteins have 38,649 residues in total and 5,050 of them are interface residues.

### Naïve Bayes classifier

We used the Naïve Bayes implementation in the Weka package from the University of Waikato, New Zealand [28,29]. For each input target residue, the classifier produces a Boolean output (with 1 denoting an interface residue and 0 denoting a non-interface residue). The Naïve Bayes classifier assumes independence of the attributes given the class. The Naïve Bayes classifier performs as well as more sophisticated methods on many classification tasks [30]. For an input *X *= *x*_1 _*x*_2 _,...,*x*_*n *_, a Naïve Bayes classifier assigns it a class label *c *by optimizing the posterior: c=arg⁡max⁡cP(c|X=x1x2...xn)=arg⁡max⁡cP(c)∏i=1nP(xi|c)
 MathType@MTEF@5@5@+=feaafiart1ev1aaatCvAUfKttLearuWrP9MDH5MBPbIqV92AaeXatLxBI9gBaebbnrfifHhDYfgasaacH8akY=wiFfYdH8Gipec8Eeeu0xXdbba9frFj0=OqFfea0dXdd9vqai=hGuQ8kuc9pgc9s8qqaq=dirpe0xb9q8qiLsFr0=vr0=vr0dc8meaabaqaciaacaGaaeqabaqabeGadaaakeaacqWGJbWycqGH9aqpcyGGHbqycqGGYbGCcqGGNbWzdaWfqaqaaiGbc2gaTjabcggaHjabcIha4bWcbaGaem4yamgabeaakiabdcfaqjabcIcaOiabdogaJjabcYha8jabdIfayjabg2da9iabdIha4naaBaaaleaacqaIXaqmaeqaaOGaemiEaG3aaSbaaSqaaiabikdaYaqabaGccqGGUaGlcqGGUaGlcqGGUaGlcqWG4baEdaWgaaWcbaGaemOBa4gabeaakiabcMcaPiabg2da9iGbcggaHjabckhaYjabcEgaNnaaxababaGagiyBa0MaeiyyaeMaeiiEaGhaleaacqWGJbWyaeqaamXvP5wqSXMqHnxAJn0BKvguHDwzZbqegyvzYrwyUfgaiqGakiaa=bfacqqGOaakcaWFJbGaeeykaKYaaebCaeaacaWFqbGaeiikaGIaemiEaG3aaSbaaSqaaiabdMgaPbqabaGccqGG8baFcqWGJbWycqGGPaqkaSqaaiaa=LgacqGH9aqpiqaacaGFXaaabaGaa8NBaaqdcqGHpis1aaaa@7297@. In the case of two class classification (*c *∈ {0, 1}), this is equivalent to determining *c *by comparing the ratio likelihood with a parameter θ as in equation (1).

P(c=1|X=x1x2...xn)P(c=0|X=x1x2...xn)=P(c=1)∏i=1nP(xi|c=1)P(c=0)∏i=1nP(xi|c=0)>θ     (1)
 MathType@MTEF@5@5@+=feaafiart1ev1aaatCvAUfKttLearuWrP9MDH5MBPbIqV92AaeXatLxBI9gBaebbnrfifHhDYfgasaacH8akY=wiFfYdH8Gipec8Eeeu0xXdbba9frFj0=OqFfea0dXdd9vqai=hGuQ8kuc9pgc9s8qqaq=dirpe0xb9q8qiLsFr0=vr0=vr0dc8meaabaqaciaacaGaaeqabaqabeGadaaakeaadaWcaaqaaiabdcfaqjabcIcaOiabdogaJjabg2da9iabigdaXiabcYha8jabdIfayjabg2da9iabdIha4naaBaaaleaacqaIXaqmaeqaaOGaemiEaG3aaSbaaSqaaiabikdaYaqabaGccqGGUaGlcqGGUaGlcqGGUaGlcqWG4baEdaWgaaWcbaGaemOBa4gabeaakiabcMcaPaqaaiabdcfaqjabcIcaOiabdogaJjabg2da9iabicdaWiabcYha8jabdIfayjabg2da9iabdIha4naaBaaaleaacqaIXaqmaeqaaOGaemiEaG3aaSbaaSqaaiabikdaYaqabaGccqGGUaGlcqGGUaGlcqGGUaGlcqWG4baEdaWgaaWcbaGaemOBa4gabeaakiabcMcaPaaacqGH9aqpdaWcaaqaaiabdcfaqjabcIcaOiabdogaJjabg2da9iabigdaXiabcMcaPmaarahabaGaemiuaaLaeiikaGIaemiEaG3aaSbaaSqaaiabdMgaPbqabaGccqGG8baFcqWGJbWycqGH9aqpcqaIXaqmcqGGPaqkaSqaaiabdMgaPjabg2da9iabigdaXaqaaiabd6gaUbqdcqGHpis1aaGcbaGaemiuaaLaeiikaGIaem4yamMaeyypa0JaeGimaaJaeiykaKYaaebCaeaacqWGqbaucqGGOaakcqWG4baEdaWgaaWcbaGaemyAaKgabeaakiabcYha8jabdogaJjabg2da9iabicdaWiabcMcaPaWcbaGaemyAaKMaeyypa0JaeGymaedabaGaemOBa4ganiabg+GivdaaaOGaeyOpa4JaeqiUdeNaaCzcaiaaxMaadaqadaqaaiabigdaXaGaayjkaiaawMcaaaaa@8D7B@

*c *is predicted to be 1 if the ratio likelihood is greater than θ, and 0 otherwise. When a local sequence around the target residue was encoded using numeric features such as hydrophobicity, the numerical values were discretized using the discretization filter of Weka.

In a standard Naïve Bayes classifier, θ takes the value of 1. The predictions of Naïve Bayes classifier are biased in favor of the majority class when the dataset consists of unequal numbers of examples for the two classes. Hence, we trained θ to optimize classification performance on training data. We used leave-one-out cross-validation to train and test the classifier. In each round of experiment, all proteins except one were used as the training set and the remaining protein was used to test the classifier. In the training stage, the conditional probability table *P*(*x*_*i *_| *c*) and prior probability *p *(*c*) were estimated using the training set. To determine θ, the classifier was applied to the training set and different values of θ ranging from 0.01 to 1 were tested, in increments of 0.01. The value of θ for which the classifier yields the highest correlation coefficient was used to make predictions on the test set.

### Naïve Bayes classifier using only local sequence identity as input

The input to the Naïve Bayes classifier contains the identities of 2*n*+1 residues in the form of *X *= (*x*_*t*-*n *_, *x*_*t*-*n*+1 _,...,*x*_*t*-1 _,*x*_*t *_,*x*_*t*-1 _,...,*x*_*t*+*n*-1 _, *x*_*t*+*n *_), where *x_t_* is the identity of target residue, *x*_*t*-*n *_, *x*_*t*-*n*+1 _,...,*x*_*t*-1 _and *x*_*t*+1 _, *x*_*t*+*n*-1 _, *x*_*t*+*n *_ are the identities of *n *residues on each side of the target residue. Different values of *n *from 1 to 10 were tried and the best performance was obtained when *n *= 4 (corresponding to a window size of 9). A training example is an ordered pair (*X*, *c*), where *c *∈ {0, 1}. 1 indicates that the target residue (the residue in the center of the input window) is an interface residue and 0 indicates that target residue is not an interface residue. For a test example *X*, the classifier outputs 1 (i.e., *X *is predicted to be an interface residue) or 0 (i.e., *X *is predicted to be a non-interface residue) as the class label of *X*.

### Naïve Bayes classifier using additional inputs

Relative solvent accessibility (rASA), sequence entropy, secondary structure, electrostatic potential and hydrophobicity were considered. When a feature of the target residue is added into the input of amino acid identities of residues in a 9-residue window, the input to the classifier is encoded as *X *= (*x*_*t*-*n *_, *x*_*t*-*n*+1 _,...,*x*_*t*-1 _,*x*_*t *_,*x*_*t*+1 _,...,*x*_*t*+*n*-1 _, *x*_*t*+*n *_*, f*_*t *_), with *f*_*t *_standing for the corresponding feature of the target residue (e.g., sequence entropy, hydrophobicity, etc.), and *x*_*i *_denotes the amino acid identity of the corresponding position within the sequence window. When a feature other than residue identity of the input window (i.e., the target residue and its sequence neighbors) is used to encode the local sequence around the target residue, the input to the classifier has the form of *X *= (*f*_*t*-*n *_, *f*_*t*-*n*+1 _,...,*f*_*t*-1 _, *f*_*t *_, *f*_*t*+1 _,...,*f*_*t*+*n*-1 _, *f*_*t*+*n *_), where *f*_*i *_is the corresponding feature (e.g., hydrophobicity) of the residue *i*.

The relative solvent accessible surface area (rASA) of each residue (in the absence of DNA) was computed using NACCESS [27]. Entropy of each sequence position (the sequence entropy for the corresponding column in multiple of the multiple sequence alignment) was extracted from the HSSP database [31]. The sequence entropy is normalized to the range of 0–100, with lower entropy values corresponding to more conserved sequence positions. Secondary structure for each residue was extracted from the PDB database [25]. Electrostatic potential for each atom was calculated using Delphi [32,33], using parameters based on the study of Jones *et al. *[10]. The electrostatic potential for each residue was calculated in a similar way as the study of Jones *et al. *[10]: the electrostatic potential of an atom is set to 0 if its solvent accessibility is less than 1Å^2 ^and the electrostatic potential of a residue is the average over all its atoms. Hydrophobicity of each residue is obtained from the consensus normalized hydrophobicity scale derived by Eisenberg *et al. *[34].

### Performance measures

Because no single performance measure provides a complete picture of performance of the classifier [35], we used a combination of *accuracy, correlation coefficient *(*CC*)*, specificity *and *sensitivity*. These measures are defined as described in Baldi *et al. *[35]. Accuracy=TP+TNN;  CC=TP×TN−FP×FN(TP+FN)(TP+FP)(TN+FP)(TN+FN);Sensitivity=TPTP+FN;Specificity=TPTP+FP
 MathType@MTEF@5@5@+=feaafiart1ev1aaatCvAUfKttLearuWrP9MDH5MBPbIqV92AaeXatLxBI9gBaebbnrfifHhDYfgasaacH8akY=wiFfYdH8Gipec8Eeeu0xXdbba9frFj0=OqFfea0dXdd9vqai=hGuQ8kuc9pgc9s8qqaq=dirpe0xb9q8qiLsFr0=vr0=vr0dc8meaabaqaciaacaGaaeqabaqabeGadaaakeaacqWGbbqqcqWGJbWycqWGJbWycqWG1bqDcqWGYbGCcqWGHbqycqWGJbWycqWG5bqEcqGH9aqpdaWcaaqaaiabdsfaujabdcfaqjabgUcaRiabdsfaujabd6eaobqaaiabd6eaobaacqGG7aWocqqGGaaicqqGGaaicqWGdbWqcqWGdbWqcqGH9aqpdaWcaaqaaiabdsfaujabdcfaqjabgEna0kabdsfaujabd6eaojabgkHiTiabdAeagjabdcfaqjabgEna0kabdAeagjabd6eaobqaamaakaaabaGaeiikaGIaemivaqLaemiuaaLaey4kaSIaemOrayKaemOta4KaeiykaKIaeiikaGIaemivaqLaemiuaaLaey4kaSIaemOrayKaemiuaaLaeiykaKIaeiikaGIaemivaqLaemOta4Kaey4kaSIaemOrayKaemiuaaLaeiykaKIaeiikaGIaemivaqLaemOta4Kaey4kaSIaemOrayKaemOta4KaeiykaKcaleqaaaaakiabcUda7iabdofatjabdwgaLjabd6gaUjabdohaZjabdMgaPjabdsha0jabdMgaPjabdAha2jabdMgaPjabdsha0jabdMha5jabg2da9maalaaabaGaemivaqLaemiuaafabaGaemivaqLaemiuaaLaey4kaSIaemOrayKaemOta4eaaiabcUda7iabdofatjabdchaWjabdwgaLjabdogaJjabdMgaPjabdAgaMjabdMgaPjabdogaJjabdMgaPjabdsha0jabdMha5jabg2da9maalaaabaGaemivaqLaemiuaafabaGaemivaqLaemiuaaLaey4kaSIaemOrayKaemiuaafaaaaa@A1BF@, where *TP****= ***the number of *true positives *(residues predicted to be DNA-binding residues that are in fact interface residues); *FP *= the number of *false positives *(residues predicted to be DNA-binding residues that are in fact not interface residues); *TN = *the number of *true negatives *(residues predicted to be non DNA-binding residues that are in fact not DNA-binding residues); *FN = *the number of *false negatives *(residues predicted to be non DNA-binding residues that are in fact DNA-binding residues); *N *= *TP+TN+FP+FN *(the total number of examples).

*Sensitivity *is the fraction of positive examples (DNA-binding residues) that are predicted as such by the classifier. *Specificity *is the fraction of positive predictions (residues predicted to be DNA-binding residues) that are actually interface residues. *Accuracy *is the fraction of overall predictions that are correct. *Correlation coefficient *measures the correlation between predictions and actual class labels.

The Receiver Operating Characteristic curve (ROC curve) is a plot of the "hit rate" (*TP*/(*TP+FN*)) versus the "false alarm rate" (*FP/*(*TN+FP*)) [35]. It shows the tradeoff between hit rate and false alarm rate when different threshold values are used for the classifier.

### Identifying PROSITE motifs in protein sequences

The PROSITE motif database was downloaded from the PROSITE [36]. Protein sequences were scanned using the ps-scan program [37] to identify motifs. Frequently matching (unspecific) patterns and profiles were omitted by setting the "-s" and "-r" options of ps-scan.

## Competing interests

The author(s) declare that they have no competing interests.

## Authors' contributions

CY carried out the computations, prepared an initial draft of the manuscript and participated in discussions and manuscript revisions. MT, and FW, and RLJ participated in discussions and manuscript reviews. DD and VH participated in experimental design, discussions, and manuscript preparation and revisions. All authors read and approved the final manuscript.

## Figures and Tables

**Table 1:** The performance of the Naive Bayes classifiers

	Identities (ID)^a^	ID + entropy ^b^
Accuracy (%)	71	78
Correlation coefficient	0.24	0.28
Specificity (%)	35	44
Sensitivity (%)	53	41

^a ^Input contains only the identities of 9 amino acid residues (the target residue and its 4 sequence neighbors on each side). ^b ^Sequence entropy of the target residue position is added as an additional input.

## References

[B1] Ghosh D, Papavassiliou AG (2005). Transcription factor therapeutics: long-shot or lodestone. Curr Med Chem.

[B2] Blancafort P, Segal DJ, Barbas CFIII (2004). Designing transcription factor architectures for drug discovery. Mol Pharmacol.

[B3] Pabo CO, Sauer RT (1992). Transcription factors: structural families and principles of DNA recognition.. Annu Rev Biochem.

[B4] Laity JH, Lee BM, Wright PE (2001). Zinc finger proteins: new insights into structural and functional diversity. Current Opinion in Structural Biology.

[B5] Lawson CL, Swigon D, Murakami KS, Darst SA, Berman HM, Ebright RH (2004). Catabolite activator protein: DNA binding and transcription activation. Current Opinion in Structural Biology.

[B6] Muller CW (2001). Transcription factors: global and detailed views. Current Opinion in Structural Biology.

[B7] Radlinska M, Kondrzycka-Dada A, Piekarowicz A, Bujnicki JM (2005). Identification of amino acids important for target recognition by the DNA:m5C methyltransferase M.NgoPII by alanine-scanning mutagenesis of residues at the protein-DNA interface. Proteins.

[B8] Griffith KL, Wolf JRE (2002). A comprehensive alanine scanning mutagenesis of the Escherichia coli transcriptional activator SoxS: identifying amino acids important for DNA binding and transcription activation. Journal of Molecular Biology.

[B9] Geyer H, Geyer R, Pingoud V (2004). A novel strategy for the identification of protein-DNA contacts by photocrosslinking and mass spectrometry. Nucleic Acids Res.

[B10] Jones S, Shanahan HP, Berman HM, Thornton JM (2003). Using electrostatic potentials to predict DNA-binding sites on DNA-binding proteins. Nucl Acids Res.

[B11] Shanahan HP, Garcia MA, Jones S, Thornton JM (2004). Identifying DNA-binding proteins using structural motifs and the electrostatic potential. Nucl Acids Res.

[B12] Tsuchiya Y, Kinoshita K, Nakamura H (2004). Structure-based prediction of DNA-binding sites on proteins using the empirical preference of electrostatic potential and the shape of molecular surfaces. Proteins.

[B13] Keil M, Exner TE, Brickmann J (2004). Pattern recognition strategies for molecular surfaces: III. Binding site prediction with a neural network. J Comput Chem.

[B14] Ahmad S, Gromiha MM, Sarai A (2004). Analysis and prediction of DNA-binding proteins and their binding residues based on composition, sequence and structural information. Bioinformatics.

[B15] Ahmad S, Sarai A (2005). PSSM-based prediction of DNA binding sites in proteins. BMC Bioinformatics.

[B16] Prediction of DNA-binding residues by PSSM and sequence homology. http://wwwnetasaorg/dbs-pssm/.

[B17] Kim JS, DeGiovanni A, Jancarik J, Adams PD, Yokota H, Kim R, Kim SH (2005). Crystal structure of DNA sequence specificity subunit of a type I restriction-modification enzyme and its functional implications. PNAS.

[B18] Jones S, Thornton JM (1997). Prediction of protein-protein interaction sites using patch analysis. J Mol Biol.

[B19] Sen TZ, Kloczkowski A, Jernigan RL, Yan C, Honavar V, Ho KM, Wang CZ, Ihm Y, Cao H, Gu X, Dobbs D (2005). Predicting binding sites of hydrolase-inhibitor complexes by combining several methods. BMC Bioinformatics.

[B20] Yan C, Dobbs D, Honavar V (2004). A two-stage classifier for identification of protein-protein interface residues. Bioinformatics.

[B21] Yan C, Honavar V, Dobbs D (2004). Identification of interface residues in protease-inhibitor and antigen-antibody complexes: a support vector machine approach. Neural Computing & Applications.

[B22] Terribilini M, Lee JH, Yan C, Jernigan RL, Honavar V, Dobbs D Prediction of RNA-binding sites in proteins based on amino acid sequence.

[B23] Berman HM, Westbrook J, Feng Z, Gilliland G, Bhat TN, Weissig H, Shindyalov IN, Bourne PE (2000). The Protein Data Bank. Nucleic Acids Research.

[B24] Wang G, Dunbrack RLJ (2003). PISCES: a protein sequence culling server. Bioinformatics.

[B25] PDB derived data. ftp://ftprcsborg/pub/pdb/derived_data/.

[B26] Gene ontology annotation. http://wwwebiacuk/GOA/.

[B27] Hubbard SJ (1993). NACCESS.

[B28] Witten IH, Frank E (1999). Data mining: practical machine learning tools and techniques with Java implements.

[B29] Weka 3: Data mining software in Java. http://wwwcswaikatoacnz/~ml/weka/.

[B30] Buntine W (1991). Theory refinement on Bayesian networks: ; Los Angeles, CA..

[B31] Sander C, Schneider R (1991). Database of homology derived protein structures and the structural meaning of sequence alignment. Proteins.

[B32] Rocchia W, Alexov E, Honig B (2001). Extending the applicability of the nonlinear Poisson-Boltzmann equation: multiple dielectric constants and multivalent ions. Journal of Physical Chemistry.

[B33] Rocchia W, Sridharan S, Nicholls A, Alexov E, Chiabrera A, Honig B (2002). Rapid grid-based construction of the molecular surface for both molecules and geometric objects: applications to the finite difference Poisson-Boltzmann method. Journal of Computational Chemistry.

[B34] Eisenberg D, Weiss RM, Terwilliger TC (1984). The hydrophobicity moment detects periodicity in protein hydrophobicity. Proc Natl Acad Sci USA.

[B35] Baldi P, Brunak S, Chauvin Y, Andersen CAF (2000). Assessing the accuracy of prediction algorithms for classification: an overview. Bioinformatics.

[B36] Hulo N, Bairoch A, Bulliard V, Cerutti L, De Castro E, Langendijk-Genevaux PS, Pagni M, Sigrist CJA (2006). The PROSITE database. Nucl Acids Res.

[B37] ps_scan program. ftp://caexpasyorg/databases/prosite/tools/ps_scan/.

[B38] Martz E (2002). Protein Explorer: easy yet powerful macromolecular visualization. Trends Biochem Sci.

